# Head and Neck Cancer Segmentation in FDG PET Images: Performance Comparison of Convolutional Neural Networks and Vision Transformers

**DOI:** 10.3390/tomography9050151

**Published:** 2023-10-18

**Authors:** Xiaofan Xiong, Brian J. Smith, Stephen A. Graves, Michael M. Graham, John M. Buatti, Reinhard R. Beichel

**Affiliations:** 1 Department of Biomedical Engineering, The University of Iowa, Iowa City, IA 52242, USA; 2 Department of Biostatistics, The University of Iowa, Iowa City, IA 52242, USA; 3 Department of Radiology, The University of Iowa, Iowa City, IA 52242, USA; stephen-a-graves@uiowa.edu (S.A.G.);; 4 Department of Radiation Oncology, University of Iowa Hospitals and Clinics, Iowa City, IA 52242, USA; 5 Department of Electrical and Computer Engineering, The University of Iowa, Iowa City, IA 52242, USA

**Keywords:** head and neck cancer, segmentation, FDG PET, CNN, Vision Transformer

## Abstract

Convolutional neural networks (CNNs) have a proven track record in medical image segmentation. Recently, Vision Transformers were introduced and are gaining popularity for many computer vision applications, including object detection, classification, and segmentation. Machine learning algorithms such as CNNs or Transformers are subject to an inductive bias, which can have a significant impact on the performance of machine learning models. This is especially relevant for medical image segmentation applications where limited training data are available, and a model’s inductive bias should help it to generalize well. In this work, we quantitatively assess the performance of two CNN-based networks (U-Net and U-Net-CBAM) and three popular Transformer-based segmentation network architectures (UNETR, TransBTS, and VT-UNet) in the context of HNC lesion segmentation in volumetric [F-18] fluorodeoxyglucose (FDG) PET scans. For performance assessment, 272 FDG PET-CT scans of a clinical trial (ACRIN 6685) were utilized, which includes a total of 650 lesions (primary: 272 and secondary: 378). The image data used are highly diverse and representative for clinical use. For performance analysis, several error metrics were utilized. The achieved Dice coefficient ranged from 0.833 to 0.809 with the best performance being achieved by CNN-based approaches. U-Net-CBAM, which utilizes spatial and channel attention, showed several advantages for smaller lesions compared to the standard U-Net. Furthermore, our results provide some insight regarding the image features relevant for this specific segmentation application. In addition, results highlight the need to utilize primary as well as secondary lesions to derive clinically relevant segmentation performance estimates avoiding biases.

## 1. Introduction

Head and neck cancer is most commonly a squamous cell carcinoma of the upper aerodigestive track and includes the oral cavity, pharynx and larynx as well as borders between these anatomic structures. It is associated with viral infection by human papilloma virus as well as smoking and heavy alcohol use. The cancer frequently spreads to local regional lymph nodes. In head and neck cancer (HNC), F-18 fluorodeoxyglucose (FDG) PET scans are frequently used for treatment planning and quantitative assessment of disease by calculating quantitative features like the standardized uptake value (SUV), metabolic tumor volume (MTV) and total lesion glycolysis (TLG). The elevated glucose uptake is a cardinal imaging feature on PET/CT, and the degree of elevation in uptake as well as the pattern of the uptake may provide important information for both treatment planning and prognosis. Such analyses require the segmentation of lesions in FDG PET scans. Quantitative imaging features can be also used for pre-treatment outcome prediction [1]. While the current standard in clinical practice is still manual segmentation by a radiation oncologist or a trained expert, a number of segmentation methods have been developed with the goal to simplify this process and increase segmentation consistency. These methods can be roughly classified into threshold-based methods and more advanced algorithm-based methods [2]. Specifically for HNC, there are many advanced algorithm-based methods such as graph-cut [3], k-nearest neighbor (KNN) [4], Markov random fields [5], and decision trees [6]. Recently, a growing number of approaches are utilizing deep learning methods with the promise of greatly improved performance [7], and a number of deep learning-based methods have been proposed [8,9]. A direct comparison of deep learning methods against the classical machine learning methods can be found in the work by Groendahl et al. [10], which shows that a 2D U-Net [11], a popular and powerful variant of a convolutional neural network (CNN), outperforms all other classical methods. A recent summary of state-of-the-art deep learning-based methods is provided by the “HECKTOR” challenge [12], where most participating methods were directly or partially based on the U-Net architecture.

Aside from the success of CNNs, Transformers [13] are gaining attention from the computer vision and medical image analysis community, especially after the proposal of Vision Transformers [14] (ViT), which match or even beat CNNs in various benchmarks. In comparison, CNNs utilize convolutional kernels to derive image features, which capture local information. Thus, representing long-range feature dependencies can be an issue, especially if one wants to keep kernel sizes small to avoid increased computing times. By contrast, the ViT allows representing long-range feature dependencies by using the self-attention module, enabling pairwise interaction between patch embeddings and resulting in more effective global contextual representations. Despite its potential, the application of ViT-based models to HNC segmentation in PET scans has currently not been adequately studied, and only scant literature exists about this topic [15,16]. Sobirov et al. [15] compared a UNETR variant to U-Net-based CNNs. Li et al. [16] recently proposed a cross-modal Swin Transformer and compared it to other networks. Both works rely on utilizing a HECKTOR challenge PET-CT data set, which is exclusively focused on primary lesions.

The contribution of our work is as follows. We provide a comparison of two CNNs, including the highly successful and widely adopted U-Net [11] and a U-Net variant with an integrated attention module, as well as three Transformer-based approaches that are frequently utilized for volumetric image segmentation. In our study design, special focus has been placed on:

(a) The use of a clinically relevant HNC PET data set with 650 lesions;
(b) Inclusion of primary and secondary lesions;
(c) The use of a high-quality expert-generated ground truth;
(d) Assessment of differences regarding their significance by utilization of statistical tests.

The presented study addresses the following existing gaps.

- Machine-learning-based models like CNNs and Transformers have different inductive biases, which influence a model’s ability to learn from a given data set with certain image characteristics, data set size, etc., and also affect its performance on new data. Thus, to inform method development and optimization efforts, it is worthwhile to investigate the performance of ViT networks in the context of HNC segmentation in PET scans on a reasonably sized image data set.
- A current shortcoming of existing studies is that secondary lesions are ignored in algorithm performance assessment but are of clinical relevance. Consequently, existing performance estimates could be biased. Secondary lesions are typically harder to segment due to potentially smaller size and lower contrast, but they are used for radiation treatment planning. Furthermore, primary and secondary lesions combined are utilized to calculate indices like MTV and TLG, which are often used as quantitative image features for outcome prediction.
- There is the need for assessing performance differences regarding their statistical significance to enable meaningful conclusions, which is often omitted (e.g., [15,16]).
- To assess systematic over- or under-segmentation, adequate error metrics are required, because this knowledge is relevant for selecting segmentation methods in the context of radiation treatment.

## 2. Materials and Methods

This section is structured as follows. In Section 2.1, image data, ground truth generation, and preprocessing steps common to all networks are described. The two utilized CNN approaches are introduced in Section 2.2, and the Transformer-based methods are described in Section 2.3. Section 2.4 provides details regarding the utilized post-processing steps. Finally, in Section 2.5, the experimental setup is described.

### 2.1. Image Data and Pre-Processing

**Figure 1:**
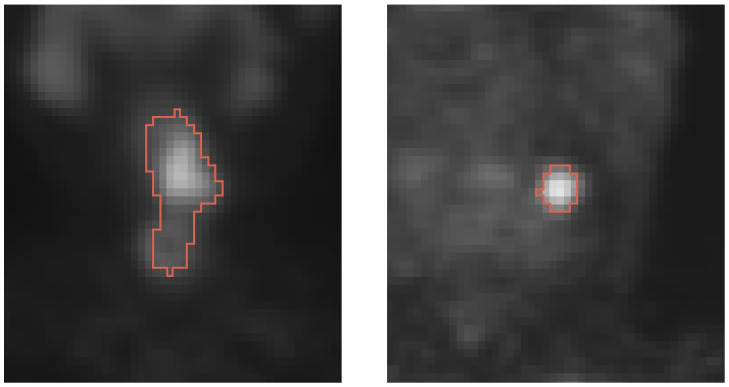
Cross-sections of a typical primary lesion (**left**) and a secondary lesion (**right**).

The availability of clinically relevant image data sets in combination with a high-quality ground truth is a major challenge when utilizing learning-based approaches such as neural networks for medical applications like lesion segmentation. To address this issue, our experiments are based on 272 HNC patient PET-CT scans from the national ACRIN-HNSCC-FDG-PET/CT trial (ACRIN 6685, secondary data analysis, data available on The Cancer Imaging Archive [17]). The scans include a total of 272 primary and 378 secondary lesions (i.e., each scan contains one primary lesion and can have up to several secondary lesions). An experienced radiation oncologist generated a ground truth for all 650 lesions by utilizing a freely available semi-automated segmentation tool [3], which was implemented in 3D Slicer [18]. All PET scans and corresponding ground truth label maps were first resampled to the median of spacings of the entire data set (2.67×2.67×3 mm) and then cropped around the center of mass of the ground truth label map of each lesion to create training, validation and test volumes of size 48×48×48 voxels, which is enough to cover the largest lesion in the data set. In this context, a tradeoff between volume size and training time as well as number of network parameters to be optimized needs to be made. If the lesion center is too close to an image boundary (<24 voxels), the cropped volume is padded using the mean of the image volume so that a consistent 48×48×48 voxel volume size is maintained. Examples of cropped volumes containing primary and secondary lesions are shown in Figure 1. For intensity normalization of PET scans, a Z-score normalization using the mean and standard deviation of each individual scan was implemented.

### 2.2. Convolutional Neural Networks (CNNs)

CNNs are currently the de-facto standard for many computer vision tasks like image classification [19]. The U-Net, introduced by Ronneberger et al. [11], is a type of CNN architecture developed for image segmentation tasks. Due to its excellent segmentation performance in a variety of applications, the U-Net architecture has been widely adopted and is frequently utilized in medical applications. The basic U-Net is described below. Transformers (Section 2.3) utilize an attention mechanism. Therefore, we also studied a U-Net variant with an attention mechanism, the U-Net with the CBAM structure (Section 2.2.2).

#### 2.2.1. U-Net

The U-Net’s architecture consists of a contracting path and an expanding path to enable the network to capture high-level as well as low-level image features. The contracting path makes use of several convolutional and pooling layers that downsample the image, while the expanding path consists of a series of convolutional and upsampling layers that upsample the image back to its original size. In addition, it makes use of skip connections to propagate information from earlier layers to deeper ones and facilitate recovering fine-grained details.

#### 2.2.2. U-Net with CBAM

**Figure 2:**
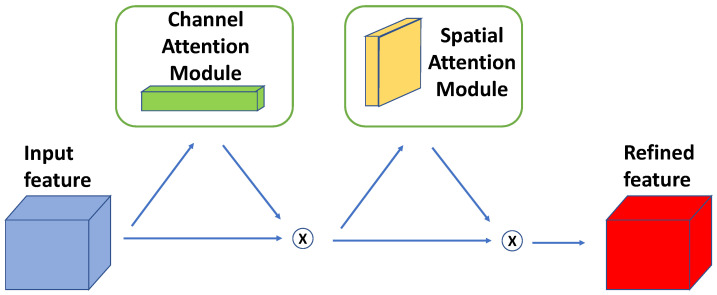
Illustration of CBAM. The module has two sequential sub-modules: channel and spatial attention. The channel attention utilizes both max-pooling and average-pooling outputs along the feature axis with a shared MLP layer, while the spatial attention utilizes the two similar outputs along the channel axis and forwards them to a convolution layer.

The convolutional block attention module (CBAM) was proposed by Woo et al. [20]. It was designed to be integrated into feed-forward CNNs and used an intermediate feature map. It infers channel and spatial attention maps, which are subsequently multiplied with the input feature map to enable adaptive feature refinement. An illustration of the CBAM is shown in Figure 2. Several approaches have been proposed to integrate CBAM or parts of it (e.g., spatial attention module) into U-Net for more robust segmentation performance [21,22,23,24,25]. These approaches are all focused on 2D segmentation problems, and the majority choose to integrate the attention module into the skip connections on the decoder part of the U-Net. One exception is the work of Guo et al. [23], who proposed a Spatial Attention U-Net (SA-UNet). At the bottleneck of a 2D U-Net, a spatial attention module is inserted to enable focusing on important features and ignoring unnecessary ones. The SA-UNet was designed for retinal vessel segmentation in 2D fundus images.

**Figure 3:**
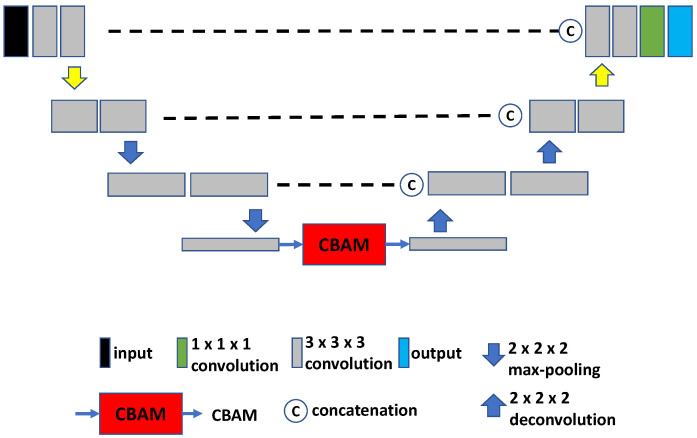
The U-Net-CBAM network integrates a CBAM into the bottleneck of a 3D U-Net.

Research on U-Net-based combined localization and segmentation has demonstrated that the bottleneck of the U-Net contains useful information for image interpretation [26]. Motivated by this insight, Xiong [27] proposed to integrate a CBAM module, consisting of a spatial and channel attention module, into a 3D U-Net architecture for the purpose of head and neck lesion segmentation in volumetric PET images. This network is depicted in Figure 3 and is referred to as U-Net-CBAM.

### 2.3. Vision Transformer-Based Models

Transformers were introduced by Vaswani et al. [13] for natural language processing. Due to their success, they were adapted to solve computer vision problems, and the Vision Transformer (ViT) proposed by Dosovitskiy et al. [14] achieved state-of-the-art performance in many applications. In medical imaging, Transformers are utilized in applications like image segmentation, classification, and detection [28,29]. Transformer utilizes a self-attention (SA) mechanism, enabling it to learn the relative importance of a single token (patch embedding) with respect to all other tokens by capturing the interaction among all tokens. Mathematically, this is carried out by transforming input $X(X\in R^{N\times D})$ into queries (Q), keys (K) and values (V) using the three separate learnable weight matrices $W^{Q}\left(W^{Q}\in R^{D\times D_{q}}\right)$, $W^{K}\left(W^{K}\in R^{D\times D_{k}}\right)$ and $W^{V}\left(W^{V}\in R^{D\times D_{v}}\right)$, where $D_{q}=D_{v}$. The input *X* is multiplied by the three weight matrices to obtain $Q=XW^{Q}$, $K=XW^{K}$, $V=XW^{V}$. Then, the SA layer output *Z* is calculated as: $Z=SA\left(X\right)=softmax\left(\frac{QK^{T}}{\sqrt{D_{q}}}\right)V$. As an expansion to Transformer, the original ViT [14] integrates the SA mechanism into computer vision for image classification by splitting images into smaller patches and flattening the patches to low-dimensional linear embeddings. The sequences of embeddings are then fed to Transformer encoders.

For a systematic comparison of segmentation performance between CNN models (U-net and U-Net-CBAM) and three representative 3D state-of-the-art Transformer-based models (TransBTS, UNETR and VT-UNet), which were proposed for medical image analysis, all networks were trained and tested using the same framework on the same data set. Note that VT-UNet represents a more pure implementation of Transformer for volumetric image data, while TransBTS and UNETR represent hybrids of a U-Net and Transformer. Each utilized Transformer-based model is briefly described below.

**Figure 4:**
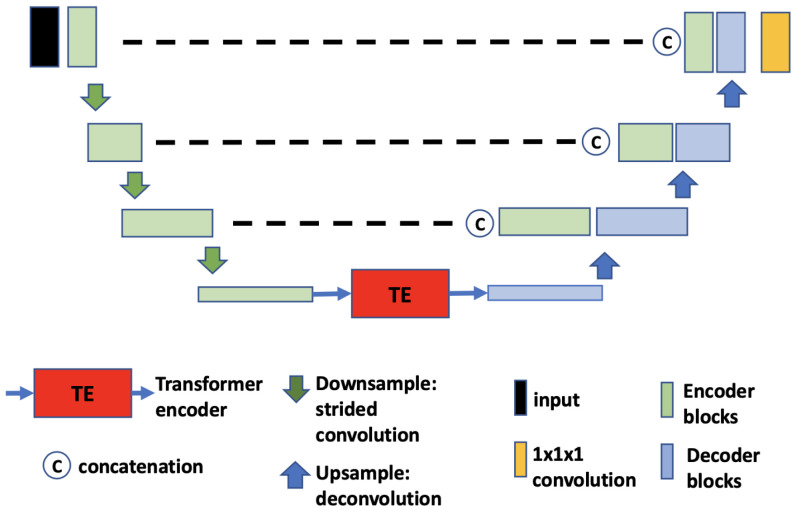
Overview of TransBTS network architecture.

**Figure 5:**
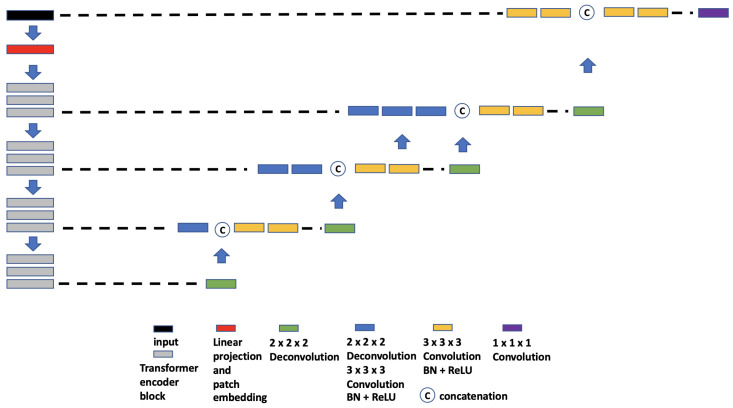
Overview of UNETR network architecture.

**Figure 6:**
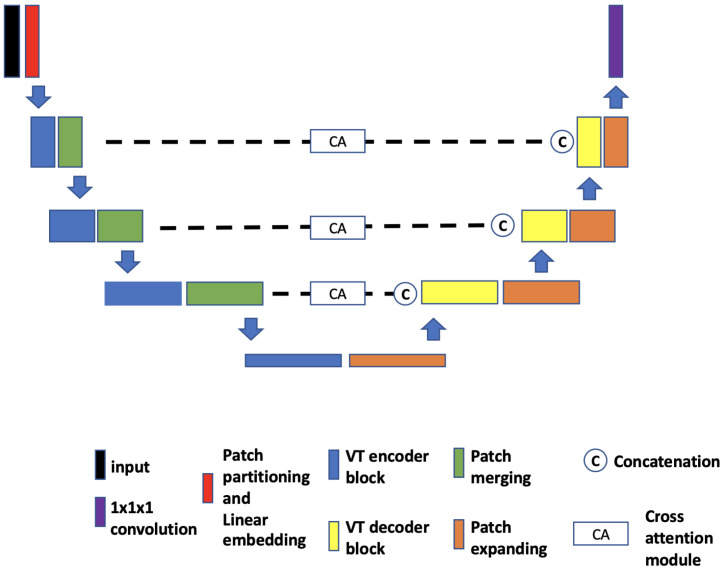
Overview of VT-UNet network architecture.

**(a)**  **TransBTS.** The core idea of TransBTS [30] (Figure 4) is to replace the bottleneck part of the 3D U-Net with a set of Transformer encoders to model the long-distance dependency in a global space [30]. The contraction–expansion structure from the U-Net is mainly utilized because splitting the data into 3D patches following the ViT makes the model unable to capture the local context information across the whole spatial and depth dimensions for volumetric segmentation. Using convolution blocks with downsampling before the Transformer encoder allows it to learn long-range correlations with a global receptive field with relatively small computational complexity.
**(b)**  **UNETR.** UNETR (Figure 5), proposed by Hatamizadeh et al. [31], is another example of the combination of CNN and ViT. In contrast to TransBTS, UNETR does not use convolution blocks and downsampling to reduce the feature size of the whole data; instead, it splits the data into 3D volume patches and then employs the Transformer as the main encoder and connects it directly to the decoder via skip connections. More specifically, as shown by Hatamizadeh et al. [31], feature representations are extracted from several different layers of the Transformer decoder and reshaped and projected from the embedding space into the input space via deconvolutional and convolutional layers. At the last Transformer layer, a deconvolutional layer is applied to upsample the feature map size by 2, and then it is concatenated with the projected Transformer output from the upper tier. The concatenated feature map is fed to consecutive convolutional layers and subsequently upsampled with a deconvolutional layer. The process is repeated until the original input resolution is reached.
**(c)**  **VT-UNet.** The VT-UNet (Figure 6) proposed by Peiris et al. [32] also built on the encoder–decoder-based U-Net architecture. However, instead of trying to combine the CNN with Transformers like TransBTS and UNETR, VT-UNet is purely based on Transformers. This is achieved by two specially-designed Transformer blocks. In the encoder, a hierarchical Transformer block is used to capture both local and global information. It is similar to Swin Transformer blocks [33]. In the decoder, a parallel cross-attention and self-attention module is utilized. It enables creating a bridge between queries from the decoder and keys/values from the encoder. This architecture enables preserving global information during the decoding process [32]. More specifically, the Swin Transformer-based encoder expands the original 2D Swin Transformer to create a hierarchical representation of the 3D input by starting from small volume patches, which are gradually merged with neighboring patches in deeper Transformer layers. Then, the linear computational complexity with input size is achieved by computing SA locally within non-overlapping windows that partition an input. In addition, a shift of the window partition between consecutive SA layers provides connections among windows and significantly enhances the model power [33]. The idea of parallelization in the decoder is to mimic the skip connections in the U-Net and serve the same purpose, enabling a connection between lower-level spatial features from lower layers and higher-level semantic features from upper layers.

### 2.4. Post-Processing

For all segmentation results, the same post-processing algorithm is applied to remove potential islands and holes. First, a connected component labeling algorithm (e.g., see [34]) is applied to the segmentation output. Then, all components except the largest are removed. Then, the resulting label map is inverted, and the two previous steps are repeated on this inverted label map (background). Finally, the resulting label map is inverted again to produce the final segmentation label map.

### 2.5. Experimental Setup

#### 2.5.1. Network Training

The hyperparameters of each network were manually optimized. For cross-validation, the 650 cropped lesion PET images and the corresponding ground truth label maps were split into three folds with roughly the same number of lesion scans so that three independent experiments can be implemented. In each of the three experiments, two folds were used for training and the other fold was used as the test data. Per experiment, a five-fold cross-validation approach was adopted, resulting in five trained models. Furthermore, all cross-validation folds were stratified per-patient. Network weights were initialized using the Kaiming initialization [35] with a normal distribution. The initial learning rate was set to 0.0001. It was reduced during network training as outlined by the “polyLR” approach [36]. The sum of binary cross-entropy (BCE) and the soft Dice loss was used as a loss function. As there can be significant differences in terms of convergence speed between CNN and Transformer models, all networks were over-trained for 3000 epochs and the final models were chosen to have the best validation loss during training. In addition, data augmentation was also applied including random rotation (range: ±45 degrees per axis), random flip and random scaling (range: ±25%).

#### 2.5.2. Network Application

To fully utilize the five-fold cross-validation setup (Section 2.5.1) in each of the three experiments, we used an ensemble approach by taking the average of the five segmentation results from five trained models.

#### 2.5.3. Performance Metrics

For segmentation performance assessment, the following error metrics were utilized: the Dice coefficient (Dice) [37], signed distance error ($d_{s}$) [38], and unsigned distance error ($d_{u}$) [38]. The selection was motivated by their relevance for clinical application. For example, the signed distance error enables assessing if there is a systematic bias, which is relevant for radiation treatment, because it might result in lesions receiving too low a dose or normal tissue receiving too high a dose. Note that for calculating $d_{s}$, segmentation surface points inside the reference are assigned a negative distance. Furthermore, a lesion segmentation is considered as failed if its Dice coefficient is at or below the 0.6 level, as manual correction of errors becomes increasingly inefficient and unpractical. Successful and failed segmentation cases are reported by providing percentages for each category. In addition, lesions sizes typically vary (Figure 1). Thus, we utilized Bland–Altman plots to assess the impact of lesion size on segmentation performance. In this context, we note that smaller lesions remain a clinical challenge since coverage is ultimately important for tumor control.

Linear mixed-effects regression models were applied to segmentation performance measures from cross-validation folds in order to statistically test the significance of mean differences between networks. Random effects were included in the models to account for correlation in means between cross-validation folds.

## 3. Results

**Figure 7:**
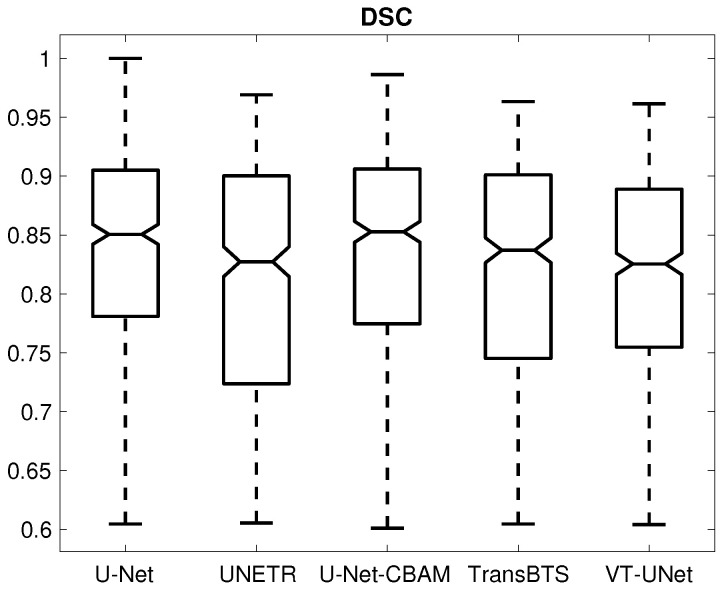
Box plot of the Dice coefficient.

**Figure 8:**
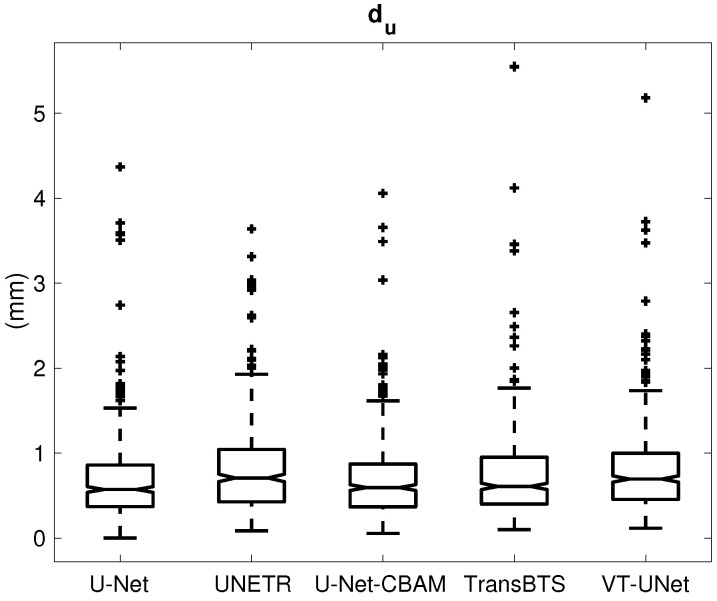
Box plot of the unsigned distance error ($d_{u}$).

**Figure 9:**
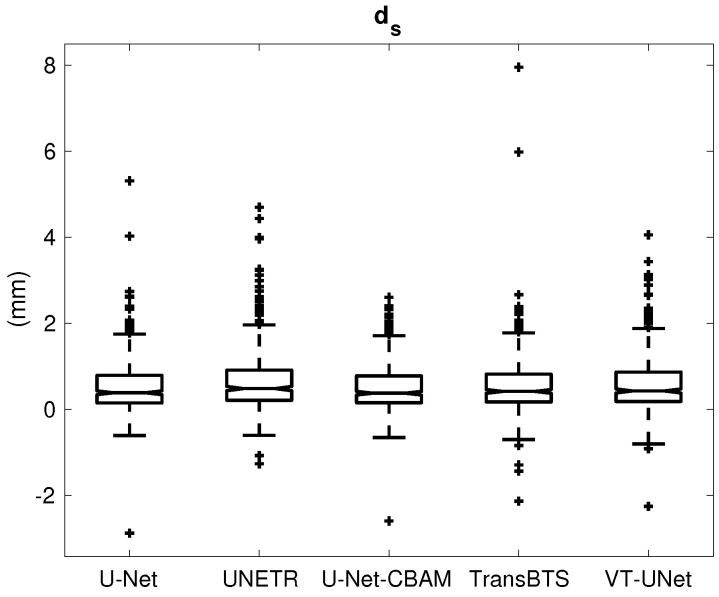
Box plot of the signed distance error ($d_{s}$).

Table 1 provides the percentage of segmentations that were deemed as successful and failed. For all successful cases, Table 2 summarizes the performance metrics for all five segmentation approaches. In addition, corresponding box plots of DSC, $d_{u}$ and $d_{s}$ are depicted in Figure 7, Figure 8 and Figure 9, respectively. As can be seen in Figure 7, medians as well as first- and third-quartile levels are lower for Transformer-based approaches when compared to U-Net variants. Based on the results for the Dice coefficient, the networks were compared to assess the statistical significance of differences. The U-Net-CBAM approach (good trade-off between DSC and percentage of successful segmentations) performed significantly better (p<0.05) than UNETR (p=0.0070) and VT-UNet (p=0.0176). By contrast, the comparisons with U-Net (p=0.9135) and TransBTS (p=0.0763) were not found to be significant. Differences in the unsigned and signed distances were not statistically significant (p>0.05). All five approaches showed a segmentation bias with the tendency to produce larger segmentations compared to the reference standard.

**Figure 10:**
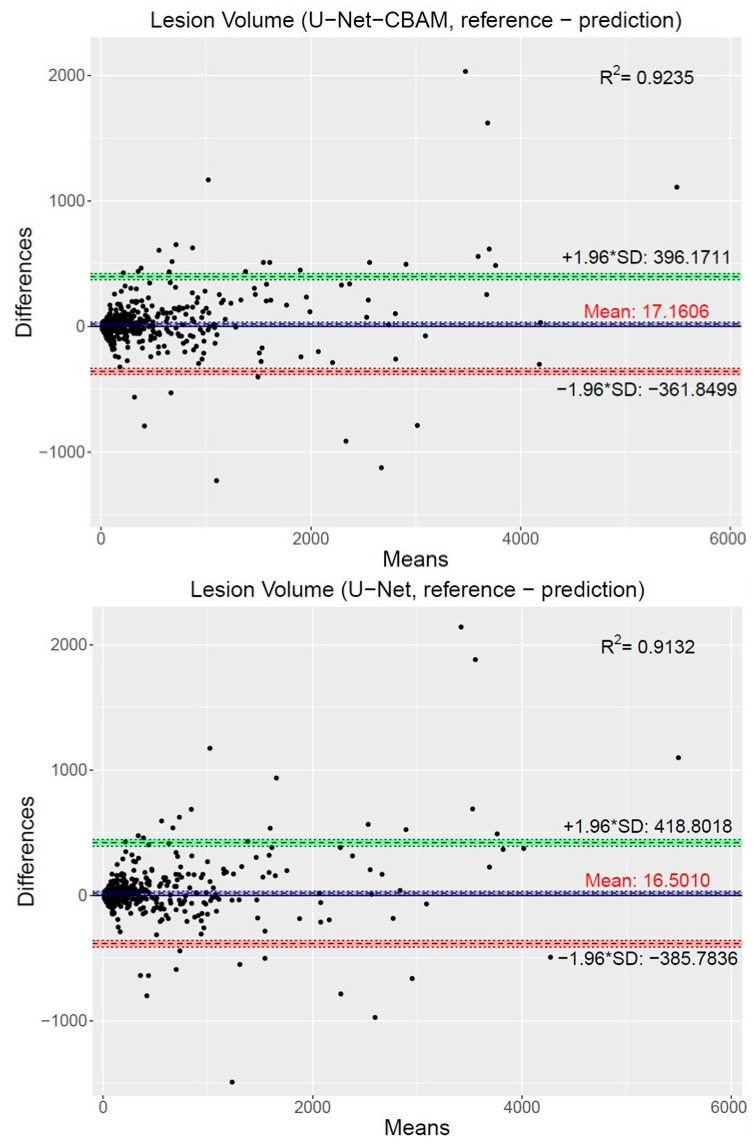
Bland–Altman plots with the representation of the limits of agreement from −1.96*SD to +1.96*SD, comparing the volume of segmentations of U-NET (top) and U-Net-CBAM (bottom) networks to the reference standard. In addition, $R^{2}$ values are shown. Note that the plots and $R^{2}$ values are based on all (successful and failed) cases.

Furthermore, we assessed the impact of lesion volume on the segmentation behavior of the top two performing networks using all segmentation results (i.e., failed and successful cases). For this purpose, the volumes of lesion segmentations generated by the U-NET and U-Net-CBAM approaches were compared to the independent reference standard by using Bland–Altman plots (Figure 10). Specifically, for each reference and generated segmentation pair, the means of the two measurements were assigned to the x-axis, and the difference between reference and prediction was assigned to the y-axis. Both methods show a bias, which is mainly driven by outliers (failed segmentations). The confidence intervals are tighter for the U-Net-CBAM approach compared to the standard U-Net. Also, R-squared ($R^{2}$) values are provided to assess the goodness-of-fit between segmentation and reference volumes (Figure 10), indicating a better fit for the U-Net-CBAM.

For U-Net and U-Net-CBAM, approximately 90% of failures occurred for secondary lesions. By looking at all segmentation results of secondary lesions (i.e., failed and successful cases), we can observe performance differences between the mean error values of U-Net-CBAM and U-Net (Dice: 0.689 vs. 0.686; $d_{u}$: 1.273 mm vs. 1.313 mm; and $d_{s}$: 1.074 mm vs. 1.084 mm). Furthermore, for CNNs and Transformers, a substantial drop of the Dice coefficient on secondary lesions (i.e., hot lymph nodes) can be observed when compared to the performance on primary lesions. Table 3 summarizes the mean relative performance difference in percentage. While TransBTS shows the smallest performance difference, we note that the overall mean Dice coefficient of TransBTS is lower compared to CNNs. Among the better performing networks, CNNs have the smallest performance difference, and U-Net-CBAM outperforms the standard U-Net. Considering these substantial differences, we conclude that it is imperative for a “real-world”, clinically relevant performance assessment of HNC segmentation approaches to consider both primary and secondary lesions, so as to avoid any potential biases in performance estimation.

**Figure 11:**
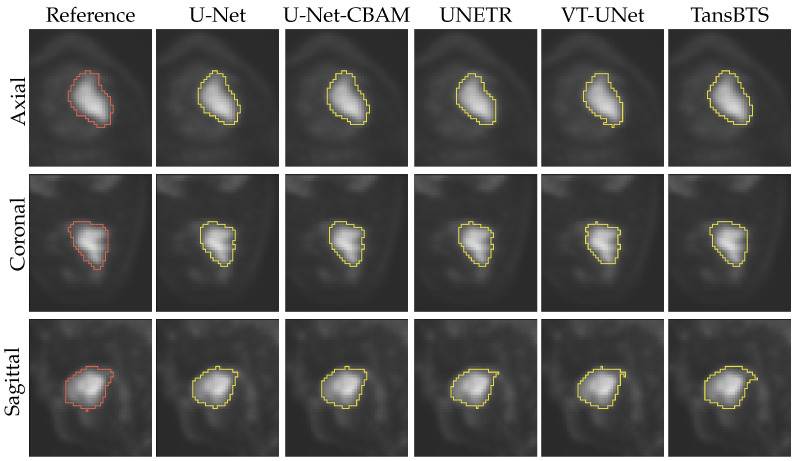
Comparison of segmentation results of a typical primary lesion from all five networks.

**Figure 12:**
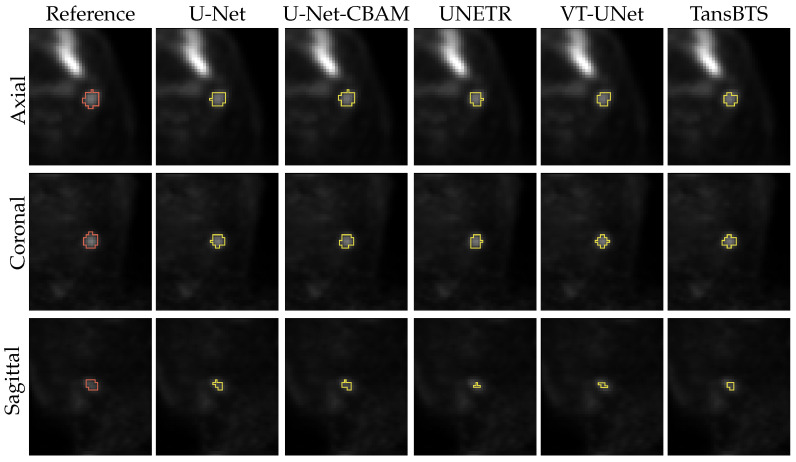
Comparison of segmentation results of a typical secondary lesion from all five networks.

**Figure 13:**
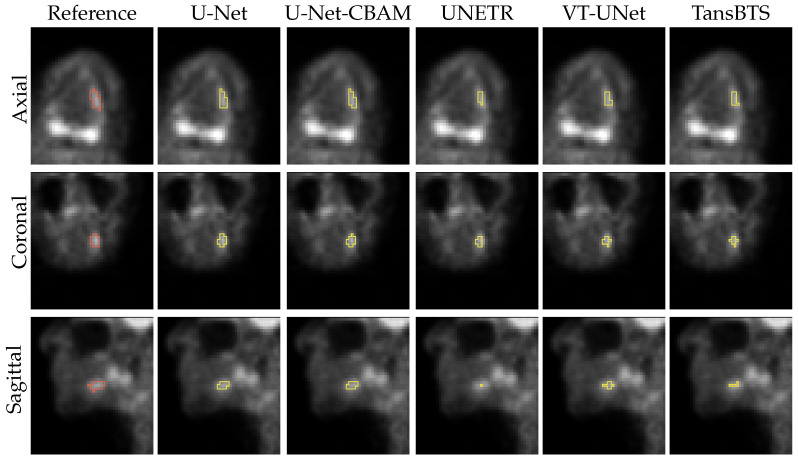
Comparison of segmentation results of a difficult primary lesion from all five networks.

**Figure 14:**
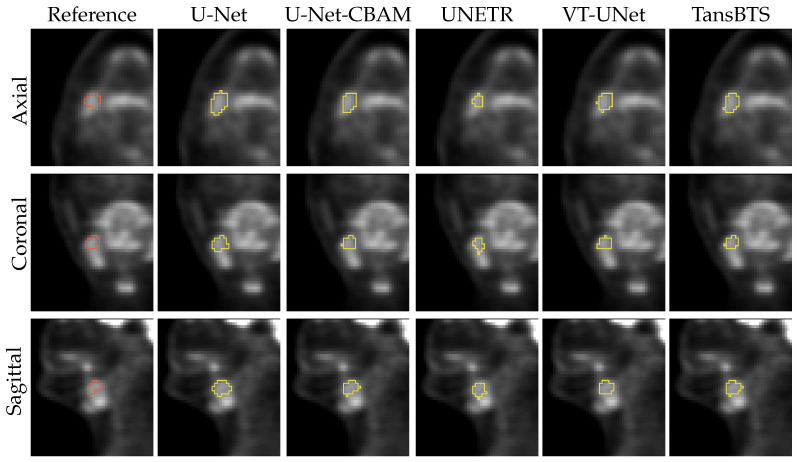
Comparison of segmentation results of a difficult secondary lesion from all five networks.

Figure 11 and Figure 12 depict typical cases of a primary and secondary lesion segmentation, respectively. In addition, Figure 13 and Figure 14 show poorly performing cases of primary and secondary lesion segmentation, respectively. Note that the lesions in good performing cases tend to have a larger size and better contrast.

## 4. Discussion

### 4.1. Segmentation Performance

On our data set, the two CNNs outperformed the three Transformer-based models investigated. The U-Net-CBAM approach represents a promising alternative to the standard U-Net. The addition of CBAM into the U-Net bottleneck resulted in better average segmentation error values across a number of error metrics investigated. By analyzing individual segmentation results, we observed that the improvements mostly resulted from better segmentation of secondary lesions (e.g., lymph nodes), which are more difficult to segment, because of the typically smaller size and lower contrast. Thus, we speculate that the refined features from the CBAM are more beneficial to the network when differentiating between foreground and background, which is more challenging yet equally important for long-term cancer control. Overall, when considering multiple clinically relevant factors like outlier percentage, Dice coefficient, distance errors, and performance on secondary lesions, we conclude that U-Net-CBAM provides the most clinically applicable and promising approach. However, in our experiments, these differences between U-Net-CBAM and U-Net were not found to be statistically significant. Thus, in future work, we plan on comparing these two networks on an enlarged data set to better assess the achievable performance differences and potential of clinical impact in correctly identifying areas at risk of tumor recurrence and hence targets for radiation therapy, and simultaneously minimizing the normal tissue identified to reduce acute and long-term sequelae of treatment.

Among the three Transformer-based models, TransBTS and VT-UNet are the most promising, and TransBTS showed the best performance. While TransBTS is a Transformer and 3D U-Net hybrid, VT-UNet is purely based on Transformers. This demonstrates the potential of the hierarchical Transformer structure and the attention bridging between encoders and decoders in semantic segmentation.

It is well-known that different network architectures have different inductive biases. For example, CNNs tend to classify images by texture and make limited use of the (global) shape [39]. By contrast, Tuli et al. [40] argue that Transformer-based approaches are more flexible, because they are not bound to utilizing convolution-type features, and have a higher shape bias, similar to human vision. Thus, we speculate that for HNC lesion segmentation in PET scans, shape seems to be less relevant, perhaps since most lesions (especially secondary lesions that represent lymph nodes) are roughly spherical or elliptical. In addition, Transformers tend to converge more slowly and require more data during training. While Transformer-based models have the potential to achieve a better segmentation performance, it seems likely that to achieve such improvements, a substantially larger training data set would be required. This is often an issue in medical applications, where available training data are typically limited and more effort is required to generate such data.

Our results show a clear segmentation performance difference for all networks when comparing results on primary and secondary head and neck lesions. The lower segmentation performance in the case of secondary lesions is expected, because they can be more difficult to differentiate from background FDG uptake yet remain clinically important areas for treatment and hence for achieving tumor control. Thus, we argue for the need to also include secondary lesions in image data sets utilized for training and testing CNNs and Transformers to avoid biases in segmentation performance estimates.

### 4.2. Current Limitations and Future Work

The goal of our work was to assess the base performance of Transformers in comparison to CNNs. Consequently, we have refrained from tweaking the investigated network structures so as to enable a fair comparison. We believe that the comparison of optimized variants is best performed in the form of challenges like HECKTOR [12]. However, the insights gained in this study are relevant for such challenges to enable clinically meaningful conclusions. Furthermore, the available training data set size also plays a role in model selection and needs to be considered. For example, for our data set size, an optimization of CNN-based methods seems to be more promising. To provide more guidance, future studies of segmentation performance’s dependence on data set size are needed.

In this work we have focused on FDG PET-driven lesion segmentation, because of its proven ability to indicate cancer regions critical for radiation treatment planning and subsequent treatment delivery. Future work will focus on assessing the impact of including CT images for lesion segmentation. Furthermore, studies are needed to assess the preference of the end users (i.e., radiation oncologists) as well as the clinical impact of using algorithms for lesion segmentation. While the preferences of individuals might vary, it will be interesting to find out whether commonalities exist or not.

One goal of our study was to assess deep-learning-based methods for their suitability to replace a graph-based segmentation algorithm of a previously published semiautomated tool designed specifically for HNC segmentation in FDG PET scans [3]. In this scenario, a trained expert provides guidance and identifies the center location of a lesion. To simulate this situation, the image volumes were cropped around the center of the target lesion. Thus, the localization of target lesions needs to be provided. Consequently, if automated segmentation of lesions is desired, a target localization approach will be necessary.

## 5. Conclusions

We have compared CNN-based networks (U-Net-CBAM and U-Net) and Transformer-based segmentation network architectures (VT-UNet, UNETR and TransBTS) in the context of HNC lesion segmentation in 3D FDG PET scans. Our results show that CNN-based architectures have an advantage in segmentation performance over the current Transformer-based network architectures in the case of the available HNC lesion FDG PET image data collected by the ACRIN 6685 clinical trial. As such, the utilized image data are quite diverse and highly relevant for clinical use, as they include primary as well as secondary lesions. Furthermore, our results provide some insight regarding segmentation-relevant image features.

## Figures and Tables

**Table 1:** Percentage of successful and failed segmentations per approach.

		U-Net	UNETR	U-Net-CBAM	TransBTS	VT-UNet
Successful segmentations	(%)	82.0	73.2	82.8	83.5	82.2
Failed segmentations	(%)	18.0	26.8	17.2	16.5	17.8

**Table 2:** Comparison of segmentation performance for successful lesion segmentations. Values are given in mean ± standard deviation format.

		U-Net	UNETR	U-Net-CBAM	TransBTS	VT-UNet
DSC	(-)	0.833 ± 0.091	0.809 ± 0.101	0.833 ± 0.092	0.819 ± 0.098	0.813 ± 0.090
$d_{u}$	(mm)	0.684 ± 0.489	0.806 ± 0.530	0.682 ± 0.464	0.740 ± 0.556	0.785 ± 0.510
$d_{s}$	(mm)	0.538 ± 0.623	0.663 ± 0.734	0.504 ± 0.550	0.559 ± 0.688	0.574 ± 0.636

**Table 3:** Mean relative difference in Dice coefficient when segmenting secondary lesions compared to primary lesions considering all (successful and failed) segmentation results.

		U-Net	UNETR	U-Net-CBAM	TransBTS	VT-UNet
Difference	(%)	−19.78	−24.57	−19.39	−7.66	−20.20

## Data Availability

PET-CT images are available at The Cancer Imaging Archive (https://www.cancerimagingarchive.net, accessed on 19 June 2020).
